# Analgesic and Antineuropathic Drugs Acting Through Central Cholinergic Mechanisms

**DOI:** 10.2174/157488911795933901

**Published:** 2011-05

**Authors:** Alessandro Bartolini, Lorenzo Di Cesare Mannelli, Carla Ghelardini

**Affiliations:** University of Florence, Department of Preclinical and Clinical Pharmacology, Viale Pieraccini 6, Florence, 50139, Italy

**Keywords:** Artemin (ARTN), analgesia, cholinergic drugs, cholinergic system, glial-derived neurotrophic factor, muscarinic receptors, neuropathic pain, nicotinic receptors.

## Abstract

The role of muscarinic and nicotinic cholinergic receptors in analgesia and neuropathic pain relief is relatively unknown. This review describes how such drugs induce analgesia or alleviate neuropathic pain by acting on the central cholinergic system. Several pharmacological strategies are discussed which increase synthesis and release of acetylcholine (ACh) from cholinergic neurons. The effects of their acute and chronic administration are described. The pharmacological strategies which facilitate the physiological functions of the cholinergic system without altering the normal modulation of cholinergic signals are highlighted. It is proposed that full agonists of muscarinic or nicotinic receptors should be avoided. Their activation is too intense and un-physiological because neuronal signals are distorted when these receptors are constantly activated. Good results can be achieved by using agents that are able to a) increase ACh synthesis, b) partially inhibit cholinesterase activity c) selectively block the autoreceptor or heteroreceptor feedback mechanisms.

Activation of M_1_ subtype muscarinic receptors induces analgesia. Chronic stimulation of nicotinic (N_1_) receptors has neuronal protective effects. Recent experimental results indicate a relationship between repeated cholinergic stimulation and neurotrophic activation of the glial derived neurotrophic factor (GDNF) family. At least 9 patents covering novel chemicals for cholinergic system modulation and pain control are discussed.

## INTRODUCTION

Treatment of painful conditions has always been a major task for physicians. Ancient Egyptians treated pain syndromes with mixtures of several medicinal plants containing *Papaver somniferum* and *Cannabis indica*. These two components were later potentiated by Greek and Roman physicians by adding *Mandragora officinalis* and *Hyoscyamus niger* extracts [1]. About 1000 years before Christ, the Greek physician, Aesculapius, used “nephentes,” which was a mixture of opium, *Mandragora officinalis* and *Hyoscyamus niger*, to relieve pain. In Roman times, Celsus potentiated the analgesic effect of opium by dissolving it in a decoction of *Mandragora officinalis* and wine. Likewise, Pliny the Elder, in his* Historia Naturalis* reported that the juice of *Mandragora officinalis* or *Hyoscyamus niger*, administered either alone or together with *Papaver somniferum*, relieved pain of different origin**s**. Furthermore, Pliny described the bark of *Salix alba* to relieve rheumatic pain. These remedies were also used in the Middle Ages and the Renaissance [1]. The anatomist and surgeon Gabriele Falloppio again proposed to relieve pain with the ancient mixture of opium, *Mandragora officinalis* and *Hyoscyamus niger*. We now know that activation or blockade of endogenous neurotransmission systems mediated by enkephalin and/or endorphins (morphine), anandamide (*Cannabis sativa*), acetylcholine (*Mandragora officinalis*, *Hyoscyamus niger*), prostaglandins (willow bark) was used to relieve pain. In this review, the mechanisms by which the antimuscarinic components of *Mandragora officinalis* and *Hyoscyamus niger*, at very low concentrations, become indirect muscarinic agonists will be elucidated.

It is surprising that the most important current analgesic medications are almost the same used thousands of years ago. Over the centuries, progresses involved the purification of active ingredients and elucidation of their mechanism of action. More recently, some advances have been made in the treatment and prevention of neuropathic pain. This was achieved by 1) employment of novel drugs and 2) identifying their mechanisms of action.

## ROLE OF THE CHOLINERGIC SYSTEM IN ANALGESIA

The history of pain relief involving the cholinergic system begins with the discovery of Otto Loewi [2] that vagal substance (vagusstoffe) was released by parasympathetic nerve endings. Loewi identified and named this substance acetylcholine (ACh). Dale, in 1914 [3], classified the activities of the cholinergic system into two main categories: muscarinic and nicotinic. Both actions involved modulation of pain perception. Nevertheless, as documented below, the role of nicotinic receptors is secondary to the role played by M_1_ and M_2_ muscarinic receptors in analgesia. Nicotinic receptors are of primary importance for the prevention and treatment of neuropathic pain. The first indication of the antinociceptive effect of ACh and direct and indirect cholinomimetic drugs date back to the 1960s. Both direct and indirect muscarinic agonists such as oxotremorine [4-6] and its precursor tremorine [7], arecoline [8], pilocarpine [9, 10], physostigmine [11, 12], and diisopropylphosphorofluoridate (DFP) [13] induce antinociception in laboratory animals. Muscarinic-dependent analgesia is centrally mediated. Pedigo *et al.* [14] described the analgesic activity of ACh after i.c.v. injection. Bartolini *et al.* [15] reported that the M_1_ selective agonist McN-A343 increases the pain threshold when injected i.c.v. Analgesia induced by peripheral injection of AF-102B, an M_1_ agonist is blocked by i.c.v. pirenzepine, an M_1_ antagonist.

Although, analgesia via activation of muscarinic receptors was known, the therapeutic use of direct muscarinic agonists clinically was never pursued due to severe side effects such as bradycardia, hypotension, tremors, sialorrhea, diarrhea, etc. Despite these side effects, the use of *Hyoscyamus niger* and *Mandragora officinalis*, compounds able to indirectly activate the muscarinic system, ACh synthesis enhancers, catabolism inhibitors, and presynaptic receptors antagonists were used occasionally.

## ANALGESIA INDUCED BY ACH SYNTHESIS PROMOTERS

The synthesis of ACh is dependent on a continuing supply of choline and glucose or pyruvate. It is catalyzed by choline acetyltransferase (ChAT), a bisubstrate enzyme, which transfers the acetyl group of acetyl-CoA to choline forming ACh (stored in the synaptic vesicles) and CoA. The increase of CoA content in the cytoplasm directly inhibits the synthesis of ACh. Subsequently, ACh removal facilitates restarting intraneuronal synthesis of ACh [16]. Therefore, removal of CoA by its acetylation represents a pharmacological method to accelerate ACh synthesis for two reasons: (1) reduced inhibitory activity by CoA and (2) increased efficiency of ChAT due to the higher levels of acetyl-CoA. A pharmacological tool to induce cholinergic analgesia is represented by acetyl-L-carnitine (ALCAR). By delivering its acetyl group to CoA, it satisfies an ideal condition to obtain maximal ChAT activity. Acetyl-L-carnitine loses its acetyl group by means of carnitine acetyltransferase (CarAT) forming carnitine. Experiments in Swiss Webster mice and Wistar rats demonstrated that repeated i.p. administration of acetyl-L-carnitine produces a statistically significant increase in pain thresholds [17]. Acetyl-L-carnitine presumably induces analgesia by increasing ACh synthesis. On acute administration it is ineffective. After repeated administration and pre-treatment with muscarinic antagonists (atropine, pirenzepine) or ACh synthesis inhibitor (hemicolinium-3) the analgesia is reduced. It is completely absent in M_1_ knock-out animals obtained by administration of a specific antisense oligonucleotide [17]. Opioids and GABA-mimetics induced analgesia in M_1_ knock-out animals [18], further confirming the cholinergic mechanism of action of acetyl-L-carnitine. Dolezal and Tucek [19] demonstrated that tritium-labeled acetyl-L-carnitine produces [^3^H]ACh in rat caudate slices. Furthermore, White and Scates [20] demonstrated that [^14^C]ACh was formed directly proportional to the amount of [^14^C] acetyl-L-carnitine present in synaptosomal membrane preparations Fig. (**1**). 

Finally, Imperato *et al.* [21] demonstrated, by microdialysis, that acetyl-L-carnitine increases the release of ACh from striatum and hippocampus of freely moving rats. The analgesic effects of acetyl-L-carnitine, observed in laboratory animals have been demonstrated also in humans. Acetyl-L-carnitine is effective in reducing pain caused by traumatic injury, diabetes, and viral infections. Intramuscular chronic treatment with acetyl-L-carnitine significantly improves the outcome of painful neuropathies or radiculopathies [22, 23].

A beneficial effect of acetyl-L-carnitine has also been reported in the treatment of symptomatic diabetic neuropathy [24-27] and in the treatment of pain in distal symmetrical polyneuropathy related to HIV infection [28]. Hence by increasing ACh synthesis and release, acetyl-L-carnitine potentiates the activation of both muscarinic and nicotinic receptors. Analgesia is thus induced by stimulation of muscarinic (M_1_) receptors (a symptomatic effect) while stimulation of nicotinic receptors has an anti-neuropathic therapeutic effect (refer to section on neuropathic pain). Numerous precursors of the ACh synthesis such as choline, phosphatidylcholine (lecithin), alfa-glyceryl-phosphorylcholine (choline alphoscerate) and cytidine-5´-diphos-phocholine have been proposed to potentiate ACh synthesis. Nevertheless, so far, no data has been reported on analgesic activity induced by the administration of these drugs suggesting that an increase in acetyl groups is a more effective therapeutic strategy.

## ANALGESIA INDUCED BY CHOLINESTERASE INHIBITORS

In 1969, Harris *et al.* [11] described the analgesic effect of physostigmine (eserine) in laboratory animals. However as early as in 1940 some investigators already realized that anticholinesterase agents have antinociceptive activity since they were able to enhance the analgesic action of opiates [29-31]. Later, we demonstrated [32] that the analgesic effect of the cholinesterase inhibitor huperzine is antagonized by adequate concentrations of scopolamine (0.1mg kg^-1^ i.p.). Not only is this due to the activation of muscarinic receptors, but also this compound, as well as physostigmine [18], has a central mechanism of action. Analgesia can be prevented by the i.c.v. administration of an aODN (antisense oligonucleotide) against M_1_ receptors [32]. It should be noted that eseroline [33-37], a compound structurally related to physostigmine, is a potent analgesic. Eseroline has two different mechanisms of actions. It produces selective blockade of acetylcholinesterases (no activity on the pseudocholinesterases) and stimulation of opioid receptors. The chemical structure of eseroline is almost identical to that of physostigmine except for the lack of the methylcarbamyl group. The lack of this group prevents eseroline from interacting with pseudocholinesterases and, therefore, has milder effects than physostigmine. Eseroline can be administered at doses about 100 times higher (10 mg kg^-1^) than physostigmine. At these concentrations eseroline activates both opioid receptors directly and indirectly muscarinic effects through the inhibition of acetylcholinesterase. By contrast, physostigmine is only able to activate the cholinergic system since at therapeutic concentrations (0.1mg kg^-1^) it does not interact with opioid receptors.

By comparing the chemical structures of the three molecules eseroline, physostigmine and morphine, one can observe that they share the same spatial configuration required for an interaction with opioid receptors Fig. (**2**).

These molecular similarities suggest why the induced analgesia by eseroline is mediated by both the cholinergic and opioid systems. The dual mechanism of action of eseroline is well documented by experiments performed on electrically stimulated longitudinal fibers of guinea pig ileum Fig. (**3**). Concentrations ranging from 0.2 and 0.8µg/ml, eseroline dose-dependently inhibits contractions whereas at higher concentrations (ranging from 1.6 and 8.3µg/ml) it has progressively decreasing inhibitory effects.

This experiment indicates that at low doses the opioid component of eseroline prevails. At higher concentrations inhibition of acetylcholinesterase counteracts the opioid-mediated inhibitory effect. To further support this hypothesis the administration of naloxone, by blocking the opioid component, unmasked the stimulatory effect produced by the inhibition of acetylcholinesterases.

It is interesting to note that, in contrast to the isolated ileum in which the two mechanisms of eseroline produce opposite effects, in analgesic tests both properties contribute to its analgesic response. This pharmacologic peculiarity of eseroline allows one to consider the analgesic potency of the opioid system in comparison to the cholinergic system on different pain conditions. In the presence of inflammatory pain (writhing test) the analgesic potency of morphine is greater than that of eseroline Fig. (**4**). By contrast, in a condition of acute thermal pain, eseroline is endowed with a higher analgesic potency Fig. (**5**). 

It is well known that there are no really effective treatments for Alzheimer’s disease. However, in the early phases of the disease an improvement of the cognitive deficit can be obtained with acetylcholinesterase inhibitors endowed with low intrinsic activity. These are better tolerated due to fewer side effects. Drugs approved for this therapy are: donepezil, rivastigmine, and galantamine. These compounds, similar to other indirect cholinomimetics, are endowed with analgesic properties (Table **1**) [38-40].

Even though cholinesterase inhibitors are not commonly employed in the relief of pain due to serious side effects, some studies indicate the possibility of a clinical use of these compounds in the management of painful syndromes. Among cholinesterase inhibitors, the primary clinically-used compound is neostigmine, administered by the epidural or subarachnoidal route. This administration bypasses the blood-brain barrier. The drug can be used at low doses without any severe cholinomimetic side effects. Mainly post-operative pain can be relieved by neostigmine which is administered only as hospital treatment [41-45]. Cholinesterase inhibitors endowed with lower intrinsic activity such as donepezil can also be used to relieve other painful conditions such as migraine [38].

## ANALGESIA INDUCED BY ENHANCERS OF ACh RELEASE

As mentioned in above, the analgesic effect of direct muscarinic agonists is not of clinical use due to their side effects. It is evident that full agonist continuous stimulation of muscarinic cholinergic receptors is not physiological. This problem is less dramatic with the use of cholinesterase inhibitors. These drugs increase the synaptic content of ACh and, therefore, allow stimulation of postsynaptic receptors only during the physiological release of ACh. If the presynaptic terminal is physiologically silent, no postsynaptic stimulation occurs even in the presence of the cholinesterase inhibitor. In other words, low doses of cholinesterase inhibitors amplify physiological cholinergic neurotransmissionwithout altering it. A similar, and even better, pharmacological strategy is the use of agents to block presynaptic autoreceptors. Such blockade will amplify the cholinergic transmission by preventing auto-inhibition of further ACh release. By the use of drugs to selectively block the presynaptic autoreceptors, a condition similar to that of a volume control in an electronic amplifier can be achieved. The amplification system enhances the transmitted signal; it neither distorts nor modifies it by adding new signals. Pharmacologically, only a few compounds act through simple amplification of physiological pathways either pre- or post-synaptically. When this is achieved, compounds with a wide clinical use can be obtained. Among them currently one can cite the use of benzodiazepines. These drugs are so well accepted that they can be abused before the appearance of the physical dependence. In contrast to direct action GABAmimetics, the benzodiazepines amplify physiological GABAergic neurotransmission by interacting with an allosteric site on the GABA_A_ receptor. They are completely inactive on those neuronal circuits which, for a physiological reason, are temporarily silent. It is evident that the importance of such a therapeutic strategy is to amplify a specific physiological function rather than to indiscriminately activate or inhibit all target mechanisms of a full drug agonist or antagonist. It is possible to amplify cholinergic neurotransmission by maintaining physiological modulation by various means as follows:

### Antagonists of Muscarinic Presynaptic Autoreceptors

M_2_ receptors are located mainly presynaptically where they regulate ACh release via a negative feedback mechanisms [46, 47]. By blocking M_2_ presynaptic autoreceptors with AFDX-116, it is possible to obtain a good antinociceptive effect in mice tested on the hot-plate [48]. AFDX-116 is unable to cross the blood-brain barrier. Hence it was injected i.c.v. The best analgesic effect was obtained with a dose of 6.3 ng/mouse. Antinociception was also obtainable with the other two selective M_2_ antagonists, methoctramine [49, 50] and AQRA-741 [51]. AFDX-116 is also able to induce antinociception in rats [15]. During the first 30 min after injection, analgesia obtained with AFDX-116 is of comparable intensity to that obtained with 5mg kg^-1^ of morphine. The analgesia induced by M_2_ antagonists was without any cholinergic side effects. Pirenzepine and dicyclomine, two selective M_1_ antagonists, as well as the unselective atropine, are able to antagonize antinociception induced by AFDX-116 and methoctramine (two selective M_2_ antagonists [52, 53]). Atropine methylbromide, which is unable to cross the blood-brain barrier, and naloxone were not effective antagonists. However, cerebral ACh depletion following i.c.v. hemicholinium-3 (HC-3), completely prevents M_2_ antagonist analgesia [48, 49, 51] but does not modify the analgesic effect of direct muscarinic agonists. 

In conclusion, antagonists of muscarinic autoreceptors induce analgesia by amplifying the physiological release of ACh**. **This analgesia is blocked by antimuscarinic drugs able to antagonize the postsynaptic muscarinic receptors as well as by compounds which deplete ACh neuronal contents such as HC-3. The antagonistic effect induced by HC-3 further indicates that the mechanism of analgesic action of selective M_2_ antagonists is at a presynaptic level since these antagonists, to induce their antinociceptive activity, need the presence of neuronal ACh. The involvement of the M_2_ muscarinic receptor subtype in the induction of analgesia has also been proven in generating M_2_ muscarinic receptor knockout mice [54].

Many local anesthetics, when administered systemically in small doses, are able to induce analgesia just like AFDX-116 and methoctramine as described by Bartolini *et al.* [6]. Procaine, lidocaine and bupivacaine, administered slowly by i.v., as well as orally-administered tocainide, suppress certain types of pain in humans. These include neoplastic [55], post-operative [56-62], post-traumatic [63], neuralgic [64, 65], muscular [66, 67], adiposa dolorosa [68-71], labour [72], and pain due to administration of radiological contrast agents [73]. Local anesthetics, by blocking presynaptic muscarinic autoreceptors, increase endogenous ACh release. This in turn produces not only analgesia, but also beneficial effects on memory, attention, psychic depression (CNS effects) and prokinetic effects (mainly on PNS) [6]. In anesthetic doses, local anesthetic drugs block action potentials responsible for nerve conduction. They bind reversibly to a specific receptor site within the pore of the Na^+^ channels blocking ion movement. This action occurs with local anesthetic concentrations higher than 1 x 10^-8^ M. The analgesic and the other central and peripheral effects of local anesthetics are obtained at concentrations much lower, that is between 1 x 10^-13^ and 1 x 10^-10^ M) [6]. Hence it is difficult to attribute a role in analgesia to blockade of Na^+^ channels. We have reported that, at low concentrations (between 1 x 10^-13^ and 1 x 10^-10^ M), procaine, lidocaine and bupivacaine do not interfere with normal contractions of guinea-pig ileum myenteric plexus longitudinal muscle strips [6]. At these very low concentrations local anesthetic drugs do not interfere with Na^+^ channels mediated action potentials. However, they are able to increase neuronal ACh release by blocking the feedback mechanism mediated by muscarinic autoreceptors [6]. Indeed procaine and lidocaine are endowed with antimuscarinic effects since they are able to antagonize ACh induced contractions of guinea pig ileum without reducing contractions mediated by histamine, serotonin and BaCl_2_ [74]. Nevertheless some authors [75-78] report data showing that local anesthetic drugs can block pain by interfering with Na^+^ conductance. They speculate that a partial blockade of Na^+^ channels occurs that does not block action potential generation or propagation, but significantly increases the threshold current required for spike generation. By this mechanism, analgesia could be produced while nerve conduction remains intact. This hypothesis, in our opinion, is correct as far as neuropathic pain is concerned but not for their common analgesic effects in low doses. It is well known that neuropathic pain originates from a mechanical or biochemical lesion of nerve axons which, as a result, became electrically unstable. From the injured sensitive nerves a burst of firing occurs which induces lancinating pain. This kind of pain is sensitive to antiepileptic drugs such as carbamazepine, phenytoin, valproic acid, etc. Local anesthetic drugs by stabilizing membranes, could be active on neuropathic pain but their mechanism of action is completely different from that which we have described for analgesia. The latter is obtained at interstitial concentrations a thousand times lower. Interesting enough is the fact that the authors, who propose sodium channel partial block for the antinociceptive effect of local anesthetics, have used larger µM concentrations of local anesthetics and their experiments were performed only *in vitro*.

As already stated in the introduction, the ancient physicians Galen and Pliny the Elder used atropine-like preparations (*Mandragora* and *Hyosciamus niger*) to reduce pain. Of special interest, atropine, if used at very low doses, is able to induce analgesia in laboratory animals [79]. The pain threshold is either increased or decreased by atropine as a function of the dose injected. In the range of 1-100µg kg^-1^ s.c., atropine increases the pain threshold. In a dose of 5mg kg^-1^ it reduces the pain threshold [5]. Antinociception induced by 1-100µg kg^-1^ is completely prevented by both HC-3 and pirenzepine [79]. Therefore, very low doses of atropine behave like AFDX-116, methoctramine, AQRA-741 and local anesthetics to induce analgesia via an amplification of endogenous ACh release. The low-dose atropine effect on ACh release is also well observable in vitro on isolated guinea-pig ileum [79]. Parasympathetic fibers of the ileum can be activated either chemically by using nicotine, DMPP (dimethyl-4-phenylpiperazinium), or by electrical field stimulation. The huge difference between chemical and electrical stimulation lies in the fact that sympathetic fibers are activated only with electrical stimulation since, anatomically, sympathetic ganglia are not present in the ileum which, on the contrary, contain para-sympathetic ganglia. This is an important point since catecholamines are released by sympathetic nerves only during electrical stimulation, but not during nicotine and DMPP stimulation. Catecholamines have an inhibitory action on endogenous ACh release via α_2_ and β receptors. Very low concentrations of atropine (10^-14^M) greatly increase nicotine-evoked contractions of isolated guinea-pig ileum. In fact, nicotine, in the presence of atropine (10^-14^ M), induces an ileum contraction which is about 50% higher than without atropine. On the contrary, low doses of atropine do not modify ACh and oxotremorine effectiveness. When atropine concentration in the bath is increased up to 10^-8^ M it is not possible to induce contractions with any of the three agonists because at these higher concentrations atropine blocks postsynaptic muscarinic receptors as well. Atropine, employed at three different concentrations on electrically evoked contractions of the ileum gives the following results: an amplification of contractions at 10^-14^ M, no modification at 10^-11^ M and an antagonism at 10^-8^ M.

These *in vitro* results [79] demonstrate that atropine when used at very low concentrations between 10^-14^ and 10^-12^ M is a selective antagonist of muscarinic presynaptic auto-receptors. At higher concentrations atropine loses its potentiating activity. Both *in vitro* and *in vivo* experiments have shown that atropine has an indirect cholinomimetic activity only when used in small amounts. By using water extraction, ancient physicians could obtain only a very small quantity of *Hyoscamus niger* and *Mandragora officinarum* alkaloids. The present results provide an explanation of why it was possible to relieve pain with these remedies. It is interesting to note, that ancient Egyptians used not only morphine but also muscarinic alkaloids to relieve pain as documented in Fig. (**6**), where a priestess dispenses both *Mandragora* flowers and *Papaver somniferum* popies to a suffering man. 

Because very low doses of atropine have selective inhibitory effects only on M_2_ receptors, while usual doses have specific M_1_ and M_2_ inhibitory activity, Ghelardini *et al.* [80] deduced the dose-related behavior of atropine, might be due to different activity of its two enantiomers. They found that R-(+) and S-(-)-hyoscyamine have different stereochemical requirements on muscarinic presynaptic autoreceptors and muscarinic postsynaptic receptors Fig. (**7**) [80]. Only R-(+)-hyoscyamine was able to induce antinociception in the mouse hot-plate test, while the S-(-) enantiomer was completely inactive Fig. (**8**) [81].

Similar results were obtained using the paw-pressure test in rats and the writhing test in mice [80]. *In vitro* experiments on electrically evoked contractions of guinea-pig ileum confirmed that the effectiveness of very low concentrations of atropine is due to the R-(+) enantiomer. Atropine and its two enantiomers were able to inhibit ileum contractions at 10^-7^ M, but only the R-(+)-enantiomer was able to increase electrically-evoked contractions at 10^-13^ M just like atropine [80]. The most interesting outcome is that only one enantiomer is active on presynaptic autoreceptors. S-(-)-hyoscyamine is completely devoid of any action on our models while its enantiomer is responsible for all of the activity of racemic atropine.

### Antagonist of Cholinergic Neuronal Heteroreceptors

Enhancement of ACh release and the resulting analgesia are also produced by antagonizing two presynaptic hetero-receptors D_2_ and A_1_. Stimulation of both receptors decreases central cholinergic activation. It is well known that in Parkinson's disease the lack of striatal dopamine leaves the cholinergic system unbridled. This is responsible for many Parkinsonian symptoms such as tremors which are reduced by anticholinergic drugs. If dopamine inhibits central ACh release we can increase this release by blocking presynaptic D_2_ heteroreceptors with antipsychotic drugs such as haloperidol, raclopride, domperidone and prochlorperazine. Indeed antipsychotic drugs are used clinically for the treatment of chronic severe pain [82-85]. Recently a comprehensive review of literature analyzed 770 patients in eleven studies [86]. Quantitative analysis of these studies showed a significant reduction of pain intensity after antipsychotic administration compared to placebo. Extrapyramidal movements and sedating effects were the most frequently reported adverse effects. The authors concluded that antipsychotics might be used as an add-on therapy in pain treatments although unwanted effects have to be considered [86]. The use of antipsychotics for pain is usually restricted to hospitalized patients.

Haloperidol (0.5mg/kg i.p.) produces in the writhing test antinociception of the same intensity as that induced by M_2_ antagonists. It should be noted that the dose of 0.5 mg/kg of haloperidol does not impair mouse rota-rod performance. The antinociception induced by D_2_ antagonists (haloperidol, raclopride and prochlorperazine) is completely prevented not only by 5mg kg^-1^ of atropine and 0.1 µg/mouse i.c.v. of pirenzepine, but also by the depletion of ACh with HC-3, as shown in Fig. (**9**). The antagonism obtained with HC-3 again suggests a presynaptic mechanism of action of D_2_ antagonists. Drugs which reduce dopamine release, such as the antagonists of 5-HT_3_ receptors (ICS-205930, MDL-72222) and agonists of 5HT_1A_ receptors (sumatriptan, 8-OH-DPAT, buspirone), may also increase antinociception through a similar presynaptic mechanism [87-90]. In agreement with these results are the findings of Bianchi *et al.* [91]. They reported that 5-HT_1A_ and 5-HT_3_ agonists respectively increase and decrease ACh efflux from the cerebral cortex of freely-moving guinea-pigs.

**Fig. (10):**
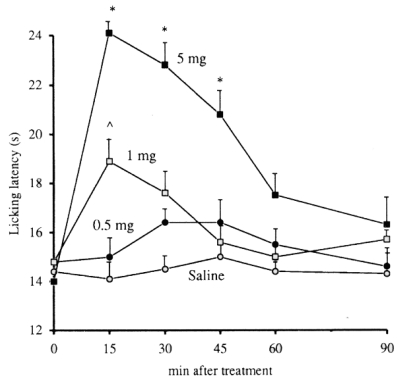
Dose-response of s.c. caffeine in the mouse hot-plate test. Doses are expressed as mg kg^-1^ [93].

Another heteroreceptor involved in inhibiting ACh release is the adenosine A_1_ receptor. In 1984, Pedata *et al.* [92], reported that 50µM caffeine (a well known A_1_ antagonist) greatly increases ACh release from electrically-stimulated rat cortical slices. It is thus not surprising that caffeine is able to induce antinociception in mice. Figure **10** shows that caffeine injected 1mg kg^-1^ s.c. produces analgesia. Pretreatment of mice either with atropine (5mg kg^-1^ i.p.) or HC-3 (1µg/mouse i.c.v.) completely blocks caffeine-induced analgesia [93]. The most likely mechanism is that by blocking A_1_ presynaptic heteroreceptors, caffeine increases endogenous ACh release which, in turn, induces analgesia via postsynaptic M_1_ receptors. It is now easy to understand why caffeine is so often a component of analgesic pills. "Cafergot" represents a famous example of such associations. The reason is not simply that of opposing the depressive effect of analgesics, but rather of improving their analgesic effects.

### Agonists of Cholinergic Neuron Heteroreceptors

As described above, it is possible to enhance cholinergic transmission by antagonism of M_2-4_ muscarinic autoreceptors as well as via D_2_ and A_1_ heteroreceptors. Enhancement of ACh endogenous release can also be obtained by stimulation of 5HT_4_ heteroreceptors. These heteroreceptors, located on the presynaptic cholinergic terminal, can amplify ACh release by a positive feed-back mechanism. Converse to the D_2_ and A_1_ heteroreceptors that regulate the ACh release by a negative feedback mechanism, these receptors induce analgesia when activated by receptor agonists. Among these agonists metoclopramide, cisapride, BIMU-1 and BIMU-8 show analgesic properties. Romanelli *et al.* [94] and Ghelardini *et al.* [95] found that the analgesia induced by these compounds is prevented by 5HT_4_ receptor antagonists such as SDZ205-557. Among the four 5HT_4_ receptor agonists, metoclopramide is the only one that has had a wide clinical use not only for its well known antiemetic and prokinetic properties, but also because it relieves painful symptoms associated with gastroenteric disorders such as gastroesophageal reflux; gastric pyrosis etc. Metoclopramide increases ACh endogenous release not only by activation of 5HT_4_ receptors, but also through D_2_ receptor antagonism. Due to its antidopaminergic activity, it can be classified between domperidone (that is unable to cross the blood-brain barrier) and haloperidol that has a marked central activity. Because of its dual mechanism of action, metoclopramide has a peculiar pharmacological profile that makes this drug particularly useful.

## ANALGESIA INDUCED BY THE CLEVER ASSOCIATION BETWEEN CHOLINESTERASE INHIBITORS AND AUTORECEPTOR-BLOCKERS

In order to increase synaptic ACh content using acetylcholinesterase inhibitors, it is necessary to inhibit the enzyme almost at 100%. With lower degrees of inhibition, the inter-synaptic content of ACh is constantly low due to the auto-regulatory effect mediated by presynaptic muscarinic receptors (negative feed-back mechanism). It is, therefore, rational to combine low doses of a cholinesterase inhibitor (physostigmine, eseroline, rivastigmine) with a M_2-4_ muscarinic autoreceptor antagonist (AFDX-116, methoctramine, R-(+)-hyoscyamine) in order to obtain greater ACh release and, consequently, analgesia with negligible side effects. In other words, with less inhibition of the acetylcholinesterase activity (about 30-50%) proportionally greater increases in the amount and half-life of synaptic ACh occurs in the presence of a blockade of the presynaptic cholinergic autoreceptor. In the absence of this blockade, the increased ACh induces an immediate activation of the autoreceptors to reduce ACh release Fig. (**11**).

In the presence of low concentrations of cholinesterase inhibitors, synaptic ACh rapidly returns to the pre-existing values. Table **2** summarizes the evidence of the potentiating effect of cholinesterase inhibitors and muscarinic autoreceptors antagonists in the mouse hot-plate test.

## SUBTYPES OF MUSCARINIC RECEPTORS INVOLVED IN ANALGESIA

There are five types of mammalian muscarinic receptors, M_1_ through M_5_, which differ structurally as determined by molecular cloning [96]. As mentioned in the section “Analgesia induced by ACh enhancers”, M_2_ and M_4_ receptors are involved in producing analgesia because of their presynaptic mechanism of action. 

These receptors reduce the pain threshold when activated [15] whereas they increase it when blocked [48-50, 97]. Conversely, the postsynaptic receptors responsible for the induction of muscarinic analgesia belong to the M_1 _subtype. Antinociception induced by muscarinic drugs has been reported to be antagonized by pretreatment with either M_1_ or M_3_ muscarinic receptor subtype antagonists, although the major role involves M_1_ receptors [15, 98-100]. Analgesia induced by administration of M_1_ selective agonists such as McN-343 and AF-102B [15] further confirms this hypothesis. Yash *et* *al*. [98] hypothesized that M_3_ receptors could also play a role in the increasing pain thresholds. These two receptor subtypes share similar pharmacological properties because both activate the same intracellular pathway through G_q/11_ proteins. We demonstrated, by means of an antisense oligonucleotide (aODN) strategy, that the only muscarinic receptor subtype involved in the induction of analgesia at a postsynaptic level is M_1_ [18]. The aODN can transiently inactivate a single gene and, therefore, can inactivate receptor functions in a more specific and selective manner than receptor antagonists. The effects on antinociceptive processes of anti-M_1_ aODN were evaluated in mice by means of the hot-plate test. A degenerate ODN (an oligonucleotide accounting for the eventual aODN nonspecific effects) was used as control. The results clearly demonstrate that M_1_ muscarinic receptors play an essential role in the modulation of pain perception. Cholinergic antinociception induced both directly, through muscarinic agonists, and indirectly, by enhancing ACh extracellular levels through cholinesterase inhibitors, is prevented, in a dose-related manner, by i.c.v. administration of an antisense to the M_1_ gene coding for the mouse M_1 _receptor [18].

## INTRACELLULAR PATHWAYS INVOLVED IN MUSCARINIC ANALGESIA

It is well established that “odd-numbered” muscarinic receptors (M_1_-M_3_-M_5_) typically couple the α subunits of the G_q/11_ family to activate phospholipase C (PLC), stimulating phosphoinositide (PI) hydrolysis [101]. In particular, reconstitution experiments (performed with purified M_1_ receptors, G protein subunits and PLC) suggest that the ß_1_ subtype of PLC serves as the primary effector for the M_1_ receptor [102]. Receptor-mediated activation of PLC results in the generation of at least two messengers, inositol-1,4,5-triphosphate (IP_3_) and diacylglycerol (DAG). The main effect of DAG is to activate protein kinase C (PKC). The effect of IP_3_ is to release Ca^++^ stored in the endoplasmic reticulum. The “even-numbered” members (M_2_-M_4_) are preferentially coupled via Gi proteins to inhibit adenylate cyclase [101]. The M_1_ muscarinic receptor subtype is responsible for postsynaptic analgesia. Galeotti *et al.* [103] elucidated the underlying intracellular mechanisms through which pain threshold is increased. Experiments were carried out in mice with acute thermal nociception. The administration of an aminosteroid: U-73122, a new phospholipase C inhibitor [104, 105], dose-dependently prevented antinoci-ception induced by physostigmine and oxotremorine [103] Fig. (**12**).

Phosphoinositide-specific PLC represents a family of isozymes found in eukaryotes composed of β, γ and δ subtypes, that cleave the polar head group from inositol lipids [106]. Among them, the isozyme PLCß_1_ has been reported to be selectively activated by M_1_ receptors [102]. In order to elucidate the role of this PLC subtype in muscarinic antinociception, an aODN complementary to the sequence of PLCß_1_ was employed. Inhibition of expression of this isozyme prevented the increase of pain threshold induced by two cholinomimetics. These results indicate PLC, and in particular the isozyme PLCß_1_, as an important intracellular effector in muscarinic analgesia [103]. The “odd-numbered” muscarinic receptors activate PLC via the α subunit of the Gq/_11_ proteins [101]. Furthermore, activation of PLCß_1_ is achieved with the help of Gα subunits of the Gq proteins [107]. Administration of aODNs against the α subunit of Gq and G_11_ proteins antagonized physostigmine and oxotremorine antinociception. These results indicate that stimulation of the PLC-mediated intracellular pathway in muscarinic analgesia requires receptor-mediated activation of Gq/_11_ transducer proteins.

As described above, PLC isozymes hydrolyze the highly phosphorylated lipid phosphatidylinositol 4,5-biphosphate generating two intracellular products: IP_3_, a universal calcium-mobilizing second messenger, and DAG, an activator of PKC. To investigate the IP_3_-mediated pathway, LiCl, an uncompetitive inhibitor of inositol monophosphatase, which regenerates inositol from inositol monophosphate, was used. The LiCl-induced inhibition depletes inositol and prevents the formation of IP_3 _[108, 109]. Animals pre-treated with LiCl showed an impaired antinociceptive response to administration of physostigmine and oxotremorine. The role of IP_3_ in muscarinic analgesia was also confirmed by dose-dependent reduction, induced by low molecular weight heparin. Heparin is a potent and selective IP_3_ receptor antagonist [110]. These results indicate the importance of IP_3_ production in the mechanism of muscarinic analgesia.

In addition to inducing IP_3_ formation, PLC causes the activation of PKC through stimulation of DAG production [106]. Pretreatment with calphostin C, a very selective, potent and membrane-permeable PKC inhibitor [111], enhanced antinociception induced by both physostigmine and oxotremorine. The administration of the PKC inhibitor also shifted to the left the dose-response curve of physostigmine. Furthermore, activation of PKC by phorbol esters, such as PMA and PDBu [112], dose-dependently prevented physostigmine and oxotremorine pain threshold increases. These data indicate that activation of PKC by cholinomimetics constitutes a significant pathway involved in negative modulation of the central muscarinic antinociceptive response. We, therefore, propose that the intracellular negative feed-back action by PKC on the central muscarinic antinociceptive pathway is necessary to maintain the basal level of pain sensitivity. The role of PLC-IP_3 _pathway in the induction of cholinergic analgesia in mice appears well established. Furthermore, the concomitant activation of PKC through DAG generation induced by cholinomimetics can partially counteract the role of PLC-IP_3_ in muscarinic antinociception.

So far, we do not know through which way PLC-IP_3_ and PKC can modulate pain perception. Recently some authors [113, 114], speculated that postsynaptic muscarinic receptors could induce analgesia either by increasing GABA release (which induces analgesia via GABA_B_ receptors) or by decreasing glutamatergic transmission (which, it is well known, to increase pain).

## ANALGESIA INDUCED BY NICOTINIC AGONISTS

Nicotine is a potent modulator of cholinergic nervous system function because of its ability to alter ion flux and neurotransmitter release which leads to various behavioral effects. Activation of nicotinic receptors elicits antinociceptive effects in a variety of species and pain tests [115-117]. Nicotine-induced antinociception appears to be a complex phenomenon that involves multiple nicotinic receptor (nAChR) subtypes depending on the pain type and sites of action. In particular α_4_ß_2_ neuronal subtypes have been implicated in thermal acute pain tests such as hot-plate and tail-flick assays [118, 119]. Knockout mice deficient in the nicotinic α_4_ß_2_ receptor subunits lack nearly all high affinity ^3^H-nicotine and ^3^H-epibatidine binding sites. They are insensitive to nicotine on the hot-plate test and display diminished sensitivity to nicotine in the tail-flick test [120]. The nAChRs exist almost exclusively on presynaptic terminals in the central nervous system and stimulate neurotransmitter release [121]. However, antinociception induced by nicotine is not due to an increase in ACh release acting on muscarinic receptors since we have demonstrated that nicotine analgesia is not modified by atropine or HC-3 pretreatment Fig. (**13**).

Contrary to the lack of effect of nicotine on ACh release *in vivo*, some authors [122, 123] have reported that nicotine is able to release ACh *in vitro* in cortical slices and synaptosomes. In our opinion the discrepancy between *in vivo* and *in vitro* experiments may depend on the high concentrations of nicotine used *in vitro* (~10^-4^ M). 

Nicotine is able to release norepinephrine *in vivo.* That could explain the antinociceptive activity of nicotine mediated via α_2_ adrenergic receptors. Li and Eisenach [121] reported that both nicotine and metanicotine induce norepinephrine release in spinal microdialysates, an effect reduced by nicotinic antagonists. Both nicotinic agonists stimulate norepinephrine release in synaptosomes. The effect of metanicotine was blocked by lower concentrations of α_4_ß_2_-nAChR antagonists. These results [121] suggest that one mechanism by which nAChR agonists produce analgesia is to stimulate spinal norepinephrine release. The release of norepinephrine is obtained via activation of the same receptor subtype (α_4_ß_2_-nAChR) responsible for nicotinic analgesia. In support of the involvement of the adrenegic α_2_ receptors in nicotine antinociception are: the antagonism exerted by yohimbine (α_2_ antagonist) [124] and the prevention by pertussis toxin (inhibitor of Gi proteins) [125] of nicotine analgesia. Since Ghelardini *et al.* [126] reported that α_2_ receptors are also responsible for analgesia induced by amitriptyline and imipramine, it appears evident antidepressants and nicotine have one common mechanism of action. Clinically, antidepressants are more used than nicotine to relieve pain. Their preference, in spite of the one common mechanism of action, could be ascribed to the fact that antidepressants increase norepinephrine concentration in the synaptic cleft by blocking reuptake mechanism while nicotine acts by activating nicotinic heteroreceptors. The first mechanism is much more physiological than the second. In the case of antidepressant drugs, the increase of neurotransmitter release takes place only when the synaptic terminal is physiologically activated. Besides nicotine, many other nicotinic agonists have been reported to increase pain threshold [127-131]. The most potent and effective is epibatidine (isolated from the skin of the frog epipedobates tricolor). Unfortunately, it is also very toxic and, therefore, has a low therapeutic index. No nicotinic agonists, excluding nicotine itself, have been found, clinically useful to date.

## CHRONIC CHOLINERGIC ACTIVATION OF N1 NICOTINIC RECEPTORS PREVENTS AND RESTORES NEUROPATHIC INJURIES

Neuropathic pain is an unpleasant, abnormal signaling associated with injury or malfunction in the nervous system. It is the main symptom of many complex disorders termed neuropathies which affect up to 7-8% of the population [132, 133]. Neuropathic pain may result from disorders of the peripheral or the central nervous system. Thus, neuropathic pain may be divided into peripheral or central or mixed (peripheral and central) neuropathic pain. Central neuropathic pain is found in spinal cord injury, multiple sclerosis, and some strokes. Aside from diabetes and other metabolic conditions, the common causes of painful peripheral neuropathies are herpes zoster infection, HIV-related neuropathies, nutritional deficiencies, toxins, remote manifestations of malignancies, genetic, and immune mediated disorders [134, 135]. Neuropathic pain is common in cancer as a direct result of compressing peripheral nerves, or as a side effect of chemotherapy, radiation injury, or surgery. As a consequence, a large percentage of patients, affected by neuropathic pain, need pharmacological treatments (carbamazepine, gabapentin, lamotrigine, pregabalin, amitriptyline, imipramine, venlafaxine, mirtazapine, nortriptyline, duloxetine, etc.). These agents are not able to prevent or revert morphological and molecular injury of tissue damage and their effects are limited to partial symptomatic relief only. Recently, we [136] reported that, by increasing ACh release and synthesis, by chronically injecting acetyl-L-carnitine in rodents, it is possible to increase artemin levels in normal and damaged areas of the nervous system Fig. (**14**). Artemin is a growth factor, belonging to the glial derived neurotrophic factor family ligands (GDFLs). They are notable for their ability to promote growth and survival of neurons. Artemin is neuroprotective and supports regeneration after nervous tissue damage [137, 138]; moreover, it is able to normalize pain thresholds. Also GDNF and other related ligands have been reported to reduce mechanical hyperalgesia and ectopic discharges within sensory neurons after nerve injury [139]. Porreca and co-workers [138,140] found that repeated artemin administration prevented pain behavior after spinal nerve ligation. Artemin and other GDNFs have anti-hyperalgesic and anti-neuropathic effects. Their characteristic actions on pain and neuron survival strongly suggests their use as therapeutical agents in neuropathy. In 2003, Gardell *et al.* [140] demonstrated that systematic administration of artemin normalized the behavioral hypersensitivity to mechanical and thermal stimuli in rat that underwent spinal nerve ligation. Artemin treatment was able to restore sensorimotor functions and improve morphological and neurochemical features of the injury state induced by spinal nerve axotomy [141] and dorsal root crush [138]. The trophic effects of artemin are in sensory neurons where its receptor GFRα3 is mainly expressed. Thus artemin could be a valuable tool to affect neuropathic pain without having broader effects on other organs and tissues [142].

Di Cesare Mannelli *et al.* [143], used a model of peripheral neuropathy described by Bennett and Xie [144], consisting of a loose ligation of the rat sciatic nerve or chronic constriction injury (CCI). They demonstrated that acetyl-L-carnitine (100 mg kg^-1^ i.p twice daily for 14 days) was able to prevent trauma-induced hyperalgesia. Under the same experimental conditions, acetyl-L-carnitine prevented regulated cell death improving a nerve apoptotic state that encompassed cytochrome C cytosolic release, activation of the cysteine protease caspase 3, up to genome fragmentation [143]. Moreover, its neuroprotective properties are also described in other animal models of neuropathy [145]. The muscarinic blocker atropine, injected to the dose of 5 mg kg^-1^ i.p., simultaneously did not antagonize the acetyl-L-carnitine anti-neuropathic effect in the paw-pressure test (right paw with CCI) Fig. (**15**).

On the contrary, as previously described above, atropine, at the same dose, significantly reduced its analgesic effects (left paw, that is the contralateral paw to CCI ones). Conversely, anti-neuropathic effect of acetyl-L-carnitine is prevented by the nicotinic antagonist mecamylamine (2mg kg^-1^ i.p. twice daily for 14 days, ipsilateral paw with CCI Fig. (**15**). This experiment shows the existence of two type of pain reverted by cholinergic activation: a) pain evoked by a mechanical stimulus applied on an healthy tissue (normal pain threshold) and b) pain evoked by the same stimulus applied on an injured tissue (reduced pain threshold). a) represents an “alarm bell” pain, the second represents a neuropathic pain. Both kind of pain are reduced by cholinegic activation, however the first is antagonized by atropine while the second is antagonized by mecamylamine. Therefore, muscarinic signal mediates analgesia likewise other analgesic drugs such as opioids and NSAID, while repeated nicotinic stimulation reverts neuropathic pain likewise antiepileptic and antidepressant drugs. Moreover, significant blockade of nicotinic receptors reduced the XIAP-related protective effect of acetyl-L-carnitine on the apoptotic state of the ligated nerve. The involvement of nicotinic receptors in acetyl-L-carnitine anti-neuropathic effect is further supported by both the prevention of neuropathic pain by chronic nicotine Fig. (**16**) in the CCI model and by the increase, following nicotine chronic treatment, of artemin mRNA Fig. (**17**) and artemin protein levels Fig. (**18**) in various parts of the peripheral and the central CNS.

These results are consistent with the hypothesis that muscarinic receptors (subtype M_1_), are involved in analgesia and the repeated activation of nicotinic receptors prevents and mitigates neuropathic injuries. The last effect appears to be mediated by growth factors of the GDNF family particularly artemin. Most relevant, the anti-neuropathic effect of acetyl-L-carnitine is completely antagonized by mecamylamine-mediated blockade of the nicotinic receptors [143]. These results differentiate drugs acting on nicotinic receptors from antiepileptics that have only a symptomatic effect on neuropathic pain.

In conclusion, acetyl-L-carnitine presents both analgesic and anti-neuropathic properties mediated by the cholinergic system. In neuropathic pain management it is clinically used without severe unwanted effects. It is able to promote ACh synthesis achieving a more physiological effect on both muscarinic and nicotinic receptors.

Clinical validation of the nicotinic acetylcholine receptors (nAChR) approach has been extensively studied. Development of nicotinic agonists for the management of moderate-to-severe pain has been limited due to cardiovascular and gastrointestinal adverse effects [146,147]. As yet, no direct nicotinic agonists have been successfully developed and approved for the treatment of neuropathic pain. Nevertheless, nicotine and nicotine receptor agonists have been demonstrated to exhibit antinociceptive, antihyperalgesic, and antiallodynic effects [146, 148, 149] across a range of preclinical models of pain, involving peripheral and central sites of action [146]. In particular the α_4_β_2_ nicotinic receptor subtype agonist ABT-594 has antinociceptive properties equal in efficacy to those of morphine across a series of diverse animal models of acute thermal, persistent chemical, and neuropathic pain states [148]. Recently, the results of a clinical phase 2 study of this compound have been published. ABT-594 (150-300µg BID) was able to significantly decrease the Pain Rating Scale in patients with diabetic peripheral neuropathic pain. However, because of adverse effects (nausea, dizziness, vomiting, abnormal dreams and asthenia) dropout rates were significantly higher than placebo indicating a limited value for possible future clinical use [150]. Other α_4_β_2_ agonists effects are blocked by mecamylamine in acute and chronic pain models in animals [146,149]. On the other hand, the α_9_α_10_ subtype, recognized in several tissue including immune cells, seems to have an opposite effect since specific antagonists can alleviate chronic pain resulting from nerve injury [151]*.*

Recent advances concerning the neuroprotective and neurotrophic effects of acute or chronic nicotine exposure are published [152]. A great deal of interest has developed that several nAChR subtypes might be involved in the neuroprotective mechanisms against neuronal damage [153]. Experimental and clinical data largely indicate long-lasting effects of nicotine and nicotinic agonists that imply both an anti-hyperalgesic and a neuroprotective role of nAChR activation, involving mainly α_7_ and α_4_β_2_ nAChR subtypes [152, 154, 155]. We have recently demonstrated that the repeated administration of the α_7_ agonist PNU-282987 (30 mg kg^-1^ once a day for 7 days and 10 mg kg^-1^ for 28 days) is able to decrease pain perception in the CCI model of peripheral neuropathy. Histological studies indicated that ligation-induced edema and macrophagic infiltrate was present. Moreover, osmicated preparations revealed a decrease in axon compactness and diameter, together with a significant loss of myelin sheaths. Repeated treatment with PNU-282987 reduced the presence of edema and macrophagic infiltrate. On nerve coronal sections, a significant increase in myelin sheath, axonal diameter and number of fibers were observable. These results strongly suggest the pivotal role of α_7_ nAChR in the neuroprotection during neuropathy [156]. 

## CONCLUDING REMARKS

Many strategies to induce analgesia involving cholinergic mechanisms are summarized. This type of analgesia has been used from the mists of time (*Mandragora*, *Hyoscyamus niger*, *Nicotiana tabacum*, *Lobelia inflata*) together with other substances (opium, *Cannabis sativa*, *Salix alba*) with different mechanisms of action. In the modern times cholinergic induced analgesia lost its importance, as a consequence of the numerous side effects. Understanding the numerous mechanisms through which this form of analgesia can be induced without aversive effects makes possible a reemergence of this therapeutic approach. All experiments performed in our laboratory [5, 6, 15, 17, 18, 33-38, 48, 49, 51, 79, 80, 87-89, 93, 136, 143] were carried out using doses of each compound that did not cause any detectable modification in laboratory animal gross behavior. All treatments did not impair motor coordination nor modify spontaneous motility or inspection activity in comparison with control groups. Moreover treatment with drugs such as ACh synthesis promoters like acetyl-L-carnitine, ACh release enhancers (R-(+)-hyoscyamine, AFDX-116, methoctramine, metoclopramide, caffeine, prochlorperazine, and low doses of local anesthetic drugs) not only did not modify rota-rod performance and other behavioral parameters, but also did not induce any detectable cholinergic symptomatology. Our studies using very small doses that produce minimal side effects indicate the value of this approach for using cholinergic agents for pain control.

## PATENTS RELATED TO CHOLINERGIC SYSTEM AS TARGET FOR PAIN TREATMENT

Cholinergic system modulation for the pharmacological management of nervous disease has provided several interesting patents. Astra Zeneca secured in 2009 (US0275574) several muscarinic receptor agonists useful in the treatment of pain, Alzheimer's disease and schizophrenia [157]. Piperidine derivatives, agonists of muscarinic receptors, have been proposed by the same company with the specific application as analgesics (WO2009034380) [158]. Also pain due to neuropathies may be treated with a muscarinic approach. Acadia Pharmaceuticals patented compounds that selectively interact with the M_1_ muscarinic receptor subtype as agonists effective in neuropathic pain (WO2004087158) [159].

No applications exist around the block of the presinaptic M_2 _subtype to enhance ACh physiological antinoceptive action. Benzocycloalkylenylamine (US6645958) [160], amino-tetralin-derivatives (US6686278) [161] and heterocyclylalkylamines (US7361648) [162] has been patented as M_2_ antagonists. All these compounds show M_3_ affinity and no authors describe them as analgesic.

In recent years, attention has been increasing for the nicotinic subtype of the ACh receptors in particular for neuropathic pain relief. Nicotinic signalling has a pivotal role both in pain sensation and neuroplasticity. Nicotinic receptor modulators are candidates to treat neuropathic pain by a neurorestorative mechanism. Peters et al, as inventors, patented diazabicyclicaryl derivatives active at nicotinic ACh receptors. These substances are useful for the treatment of peripheral and central nervous system disorders related to pain (US20100113428) [163].

A role of the immune system in the beginning and in the maintenance of neuropathic pain is well established [164]. Since the homomeric α_7_ subtype is expressed in neurons as well as in glia cells with a regulatory function it could be a target for the management of the immune component of neuropathies. Mazurov *et al.* (US20080138287) secured 3-substituted-2-(arylalkyl)-1-azabicycloalkanes as α_7_ modulators [165]. Finally, the glia-derived neurotrophic factor artemin results to be stimulated by cholinergic compounds. Biogen Idec patented a method for evaluating neuropathic pain and neurotrophic activity of a drug or a drug candidate by measuring artemin and correlated gene expression levels from skin biopsys (WO2005083125) [166].

## CURRENT AND FUTURE DEVELOPMENTS

Damage to the nervous system is the primary cause of neuropathy and chronic pain. Current pharmacological treatments for neuropathic pain are not able to prevent or reverse morphological and molecular consequences of tissue injury. Current therapies for pain relief are not able to induce tissue regeneration. The most clinically used compound gabapentin is active for pain relief but is ineffective for neuroprotection or neuroregeneration. Neuropathic pain is rapidly increasing because of many diseases such as type 2 diabetes, HIV immunodeficiency, various types of neoplastic pathologies requiring chemotherapeutic agents such as paclitaxel, oxaliplatin, cisplatin, vincristine, etc., all cause major and painful neuropathies. There exists a significant interest in developing novel pharmacological treatments to prevent and treat neuropathic pain. In this respect the artemin growth factor and the other member of the GDNF family are of great interest. Artemin is notable for its ability to promote growth and survival of neurons, after nervous tissue damage [137,138]. Artemin, contrary to neurotrophins (NGF and BDNF) is also able to normalize pain threshold. We have recently reported [136] that the ACh synthesis promoter acetyl-L-carnitine, whose patent expired long ago, is able, following repeated treatments, to display not only an antihyperalgesic effect but also neurorestorative properties and good safety profile. Clinical studies on diabetic peripheral neuropathy show that acetyl-L-carnitine reduces excessive pain sensation and improves nerve conduction velocities and regeneration [167]. Open studies involving HIV-positive patients have shown that chronic treatment with acetyl-L-carnitine ameliorates pain symptoms related to peripheral polyneuropathy [28]. Hart and colleagues [168] report that six months of oral acetyl-L-carnitine treatment result in peripheral nerve regeneration of small sensory fibers as observed from skin biopsies in patients with distal symmetrical polyneuropathy.

Future developments should involve screening for an artemin promoter and/or releaser, as well as new chemical structures capable to increase ACh synthesis and release. Alternatively, it is essential to identify specific agonists for one of the many known nicotinic subtype receptors, which are able to increase artemin levels and its release. This second strategy appears more challenging.

## CONFLICT OF INTEREST

Some scientific articles published by the authors, concerning the mechanism of action of ALCAR, were financially supported by Sigma-Tau Industrie Farmaceutiche Riunite S.p.A., Via Pontina km 30,400, I-00040 Pomezia, Rome, Italy.

## Figures and Tables

**Fig. (1):**
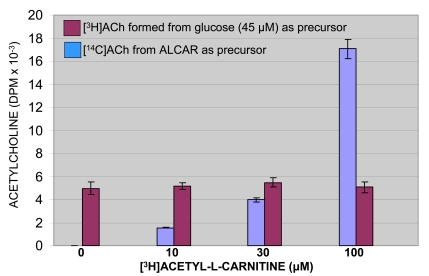
Synthesis of ACh from acetylcarnitine in a crude synaptosomal membrane preparation [^14^C]. Glucose (45 µM) was incubated for 60 min with freshly prepared extract in the absence and presence of [^3^H]acetyl-L-carnitine at concentration shown. (modified from [20]).

**Fig. (2):**
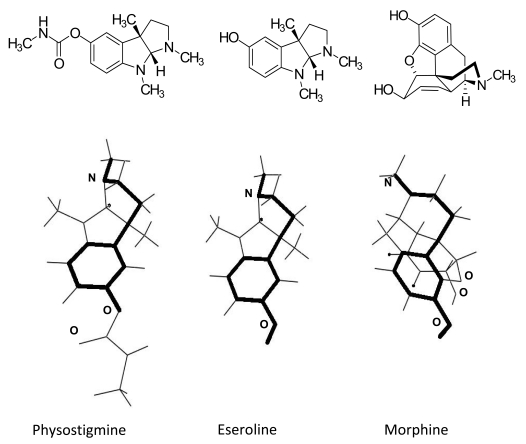
Dreiding stereomodels of chemical structures of physostigmine, eseroline and morphine.

**Fig. (3):**
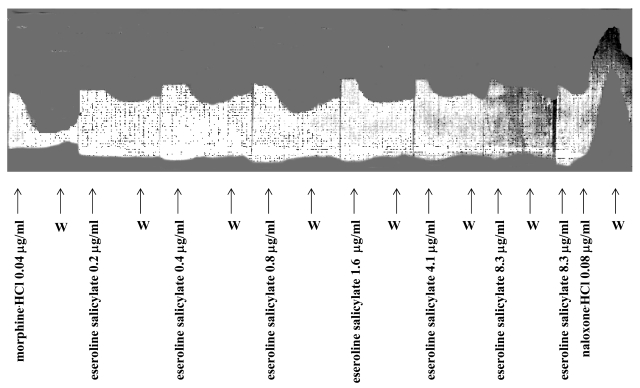
Effect of increasing doses of eseroline in comparison with morphine on the electrically evoked contractions of the myenteric plexus longitudinal muscle preparation of guinea-pig ileum and antagonism by naloxone [37].

**Fig. (4):**
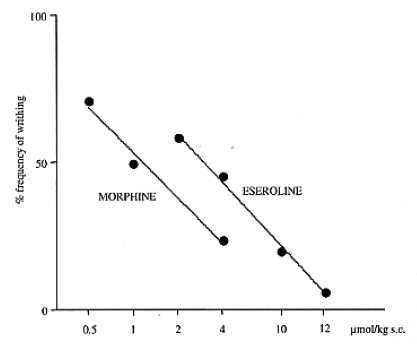
Comparison between eseroline and morphine dose-response curves in the mouse abdominal constriction test.

**Fig. (5):**
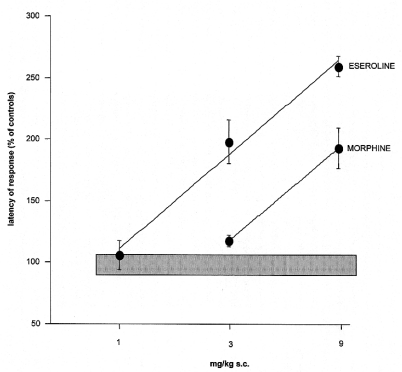
Comparison between eseroline and morphine dose-response curves in the mouse hot-plate test [40].

**Fig. (6):**
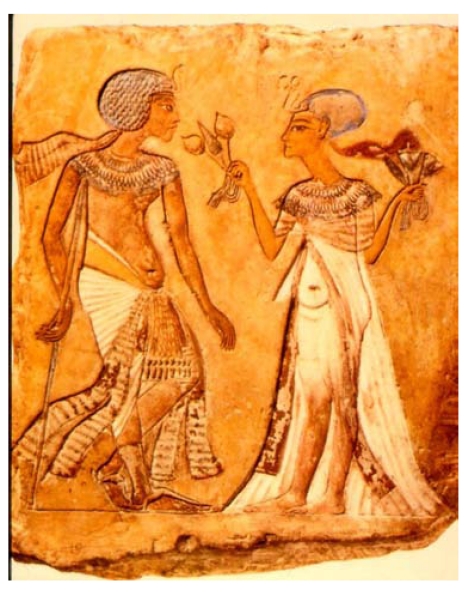
*Mandragora* flowers and *Papaver somniferum* capsules depicted on ancient friezes (Egyptian Museum of Berlin).

**Fig. (7):**
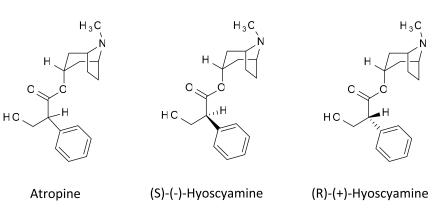
Structure of atropine and its enantiomers.

**Fig. (8):**
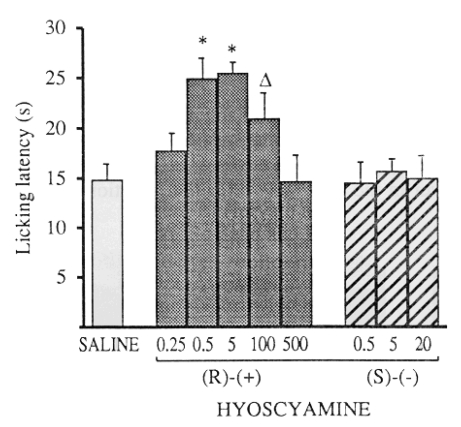
Effect of R-(+) and S-(-) hyoscyamine in the mouse hot-plate test. The test was performed 15 min after treatment. Doses are expressed as µg kg^-1^ s.c [81].

**Fig. (9):**
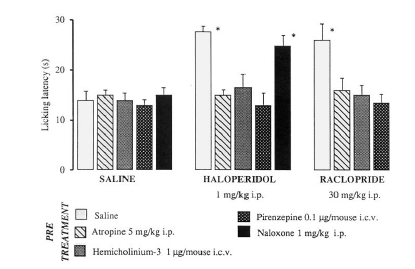
Effect of atropine, HC-3, pirenzepine and naloxone on haloperidol and raclopride antinociception in the mouse hot-plate test. The test was performed 15 min after haloperidol and raclopride injection [81].

**Fig. (11):**
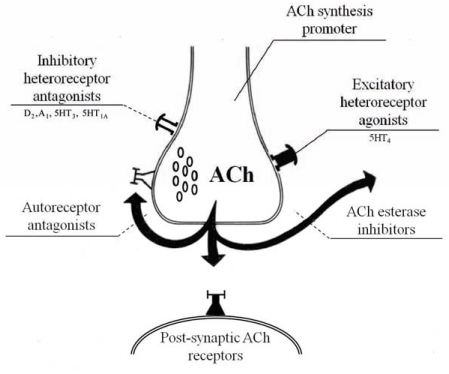
Cholinergic synapse. Synergic effect among cholinesterase inhibitors, ACh synthesis promoter, autoreceptor blockers 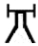 , excitatory heteroreceptor agonists 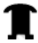, inhibitory heteroreceptor antagonists 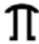. Postsynaptic ACh receptor 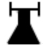.

**Fig. (12):**
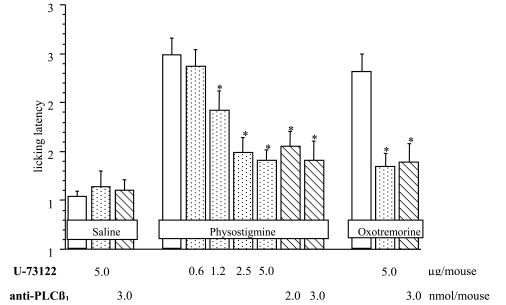
Pretreatment with U-73122 (0.6-5 µg per mouse i.c.v.) and anti-PLCβ1 (2-3 nmol per mouse i.c.v.) antagonizes physostigmine (0.1 mg kg^-1^ s.c.)- and oxotremorine (60 µg per mouse i.c.v.)-induced antinociception in the mouse hot-plate test [103].

**Fig. (13):**
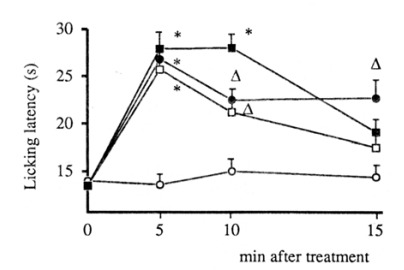
Antinociception induced by nicotine (1.5mg kg ^-1^ s.c.) in mouse hot-plate test and lack of antagonism by atropine (5mg kg^-1^ i.p.) and by HC-3 (1µg per mouse i.c.v.). Saline: open circles; Nicotine: closed squares; Atropine + Nicotine: open squares; HC-3 + nicotine: closed circles. ** P< 0.01; * P<0.05 in comparison with saline controls [81].

**Fig. (14):**
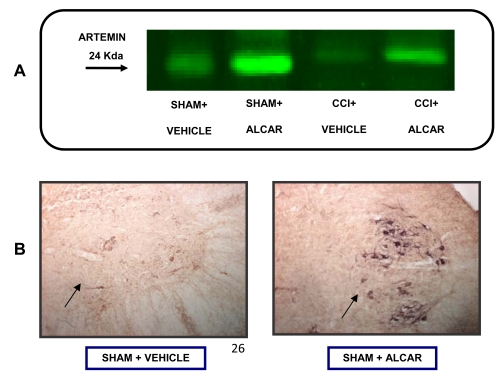
**A**): Artemin levels in spinal cord of rats detected by the Odyssey method following standard immunoblotting procedure. Rats underwent to surgical process with (CCI) or without (sham) the loose ligation of the sciatic nerve. The administration of saline or acetyl-Lcarnitine (100mg kg^-1^ i.p., twice per day) began on the day of the operation and continued for 15 days. **B**): Artemin expressed in spinal cord (immuno-histochemically detected) in saline or ALCAR treated rats (treatment time and doses as in A).

**Fig. (15):**
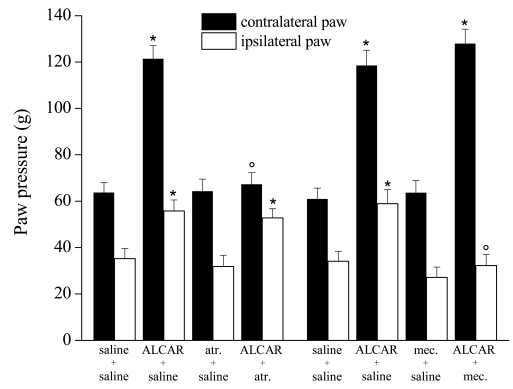
Paw pressure test: Effect of acetyl-L-carnitine in the presence of atropine (atr.) or mecamylamine (mec.). Response to noxious stimuli on the ipsilateral paw that underwent CCI respect to the contralateral un-operated paw. Effects of treatments with saline alone, acetyl-Lcarnitine (100mg/kg twice daily for 15 days), atropine (5mg/kg twice daily for 15 days) or co-administration of both. Effects of treatments with saline alone, acetyl-L-carnitine (100 mg/kg twice daily for 15 days), mecamylamine (2mg/kg twice daily for 15 days) or co-administration of ALCAR and mecamylamine. Paw Pressure test was performed 30 min after the last injection. Each value represents the mean of 2 experiments with 12 rats per group. *P < 0.01 versus control (saline + saline) rats. °P < 0.01 versus acetyl-L-carnitine + saline treated rats [143].

**Fig. (16):**
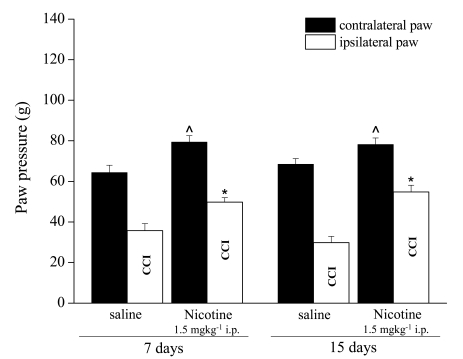
Effects of 7 days and 15 days repeated treatments of rats with nicotine (1.5 mg kg^-1^ i.p.) on paw pain threshold starting from the day of their chronic constriction injury of right sciatic nerve (CCI). Paw Pressure test was performed 10 minutes after the last nicotine or saline injection. Each value represents the mean of 2 experiments with 6 rats per group. ˆP < 0.05 and *P < 0.01 versus control (saline-treated) rats.

**Fig. (17):**
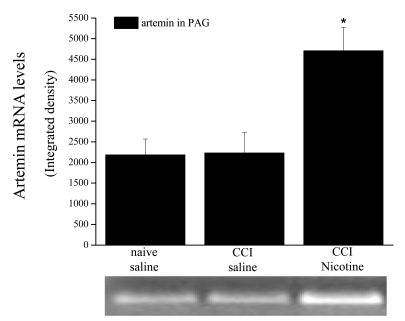
Artemin mRNA levels. Total mRNA was extracted from the periaqueductal grey matter (PAG) and RT-PCR was performed (Forward primer 5´ TAGGTGGCAACCAGCCTTG 3´; Reverse primer 5´ TGTGTCCCCCAGGTAGGT 3´). Comparison among naive, CCI and CCI-nicotine-treated rats. 1.5mg kg^-1^ nicotine was administered i.p. for 15 days starting from the day of ligation. Quantitative analysis is shown as the mean ± s.e.m of 5 different animals. *P < 0.01 with respect to naive, saline.

**Fig. (18):**
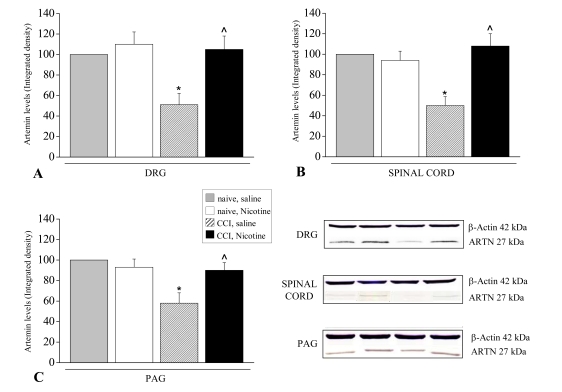
Artemin protein levels. Protein expression levels were analyzed in CCI rats in respect to naive and the effect of Nicotine (1.5 mg kg^-1^ i.p.) was evaluated after administrations repeated for 15 days. 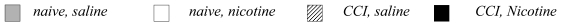 Densitometric analysis of A) ipsilateral dorsal root ganglia (DRG); B) total spinal cord; C) periaqueductal grey matter (PAG). Panel D) shows representative Western blot performed in the different areas with an artemin specific antibody; a colorimetric method was used to visualize the peroxidase-coated bands. Results are expressed as the mean ± s.e. of 5 different animals; β-actin normalization was performed for each sample. Measurements in control samples (naive, saline) were assigned a relative value of 100%. *P < 0.05, significantly different from naive, saline; ˆP < 0.05 significantly different from CCI, saline.

**Table 1.** Analgesic Effect Induced by Rivastigmine and Donepezil in the Mouse Hot-plate Test and Antagonism Exerted by Scopolamine.

	*LICKING Latency in Mice (s)*			
TREATMENT	*Before*	*After Treatment *		
	*Treatment*	30 Min	45 Min	60 Min
SALINE	16.2 ± 1.1	16.3 ± 1.4	15.5 ± 2.0	15.4 ± 1.7
SCOPOLAMINE 0.1	14.3 ± 1.2	15.0 ± 1.8	13.9 ± 2.2	15.6 ± 1.8
RIVASTIGMINE 0.5	15.9 ± 1.5	17.0 ± 1.6	16.1 ± 1.8	15.4 ± 1.6
RIVASTIGMINE 1.0	16.4 ± 1.2	25.7 ± 2.1*	25.9. ± 1.9*	24.8 ± 1.5*
RIVASTIGMINE 2.0	17.1 ± 1.8	21.6 ± 2.8*	35.9 ± 2.7*	35.5 ± 3.6*
DONEPEZIL 5	14.3 ± 1.3	33.2 ± 3.4*	25.9 ± 3.1.*	19.3 ± 2.5
DONEPEZIL 10	13.6 ± 1.4	35.1 ± 3.6*	31.5 ± 2.8*	21.7 ± 2.9*
SCOPOLAMINE +DONEPEZIL 5	14.0 ± 0.9	18.5 ± 2.6ˆ	16.4 ± 2.3ˆ	15.8 ± 1.5

The doses are expressed as mg kg^-1^ s.c. There were at least 12 mice per group.

* P < 0.01, versus saline control mice.

ˆ P < 0.01 versus Donepezil (5 mg kg ^-1^ s.c.) treated mice [40].

**Table 2.** Potentiating Effect of Physostigmine and Rivastigmine on AFDX-116 and R(+)-hyoscyamine -induced Analgesia in the Mouse Hot-plate Test.

		* Licking Latency in Mice (s)*			
TREATMENT	TREATMENT	*Before*	*After Treatment *		
		* Treatment*	15 Min	30 Min	45 Min
SALINE	SALINE	15.2 ± 1.1	14.7 ± 1.5	13.2 ± 2.0	14.6 ± 1.9
SALINE	AFDX-116	16.1 ± 1.2	23.2 ± 2.1*	20.7 ± 1.9*	16.3 ± 1.5
SALINE	R-(+)-HYOSCIAMINE	13.9 ± 0.9	24.7 ± 1.9*	22.6 ± 1.7*	17.1 ± 1.8
PHYSOSTIGMINE	SALINE	14.8 ± 1.2	25.6 ± 2.6*	21.7 ± 1.9*	19.3 ± 2.0
PHYSOSTIGMINE	AFDX-116	15.0 ± 1.1	36.5 ± 1.7*	35.2 ± 1.6*	24.0 ± 1.7*
PHYSOSTIGMINE	R-(+)-HYOSCIAMINE	14.3 ± 1.5	39.1 ± 2.4*	36.2 ± 2.3*	26.7 ± 2.8*
RIVASTIGMINE	SALINE	14.3 ± 1.1	26.2 ± 2.1*	22.5 ± 1.2*	17.5 ± 1.2
RIVASTIGMINE	AFDX-116	13.2 ± 1.7	38.7 ± 2.8*	35.5 ± 2.6*	22.9 ± 1.8*
RIVASTIGMINE	R-(+)-HYOSCIAMINE	14.5 ± 1.2	37.0 ± 3.2*	34.6 ± 2.9*	26.5 ± 1.6*

AFDX-116 (0.1μg i.c.v.) and R-(+)-hyoscyamine (10μg i.c.v.) were administered 30 min after rivastigmine (0.75mg kg^-1^ s.c ) and physostigmine (0.075mg kg^-1^ s.c). There were at least 12 mice per group.

* P < 0.01, versus control mice.

ˆ P < 0.01 versus saline AFDX/R-(+)-hyosciamine [40].

## ABBREVIATIONS

ACh: = Acetylcholine
ALCAR: = Acetyl-L-carnitine
aODN: = Antisense Oligodeoxynucleotide
ARTEMIN: = Artemin
CCI: = Chronic constriction injury
ChAT: = Choline acetyltransferase
CNS: = Central Nervous System
CarAT: = Carnitine acetyltransferase
DAG: = Diacylglycerol
DFP: = Diisopropyl-phosphorofluoridate or Diisopropyl fluorophosphate
DMPP: = Dimethyl-4-phenylpiperazinium
GABA: = Gamma-Aminobutyric acid
GDNF: = Glial cell line-derived neurotrophic factor
GFLs: = Glial cell-line derived neurotrophic factor family ligands
HC-3: = Hemicholinium-3
HIV: = Human immunodeficiency virus
IP_3_: = Inositol trisphosphate
nAChR: = Nicotinic receptor
PAG: = Periaqueductal grey matter
PI: = Phosphatidylinositol
PKC: = Protein kinase C
PLC: = Phospholipase C
PNS: = Peripheral nervous system
XIAP: = X-linked Inhibitor of Apoptosis Protein
